# Indiana State Board of Dental Examiners

**Published:** 1888-03

**Authors:** P. G. C. Hunt, R. Van Valzah, S. T. Kirk, E. J. Church, M. H. Chappell

**Affiliations:** President, Indianapolis; Terre Haute; Kokomo; LaPorte; Knightstown


### CORRESPONDENCE.

### Indiana State Board of Dental Examiners.

KNIGHTSTOWN, IND., March 3d, 1888.

*Dear Doctor:—*

The dental law of 1887, of this State, has been tested on the case of George Wilkins, of Marion, Grant County. Tried in the December term, 1887, of Grant Circuit Court, and the Circuit Judge held the law constitutional, found the defendant guilty and fined him $20.00 with cost. Wilkins appealed. The case was before the Supreme Court and decision made March 2, 1888. "The act of March 7, 1887, regulating dentistry, does not conflict with the Constitution in any respect, and is valid," etc.

The Board of Examiners at their first session, July 1st to the 5th, 1887, gave notice that they would not receive diplomas, as conclusive, from certain colleges, from the fact that the Indiana Board was a member of the National Board of Dental Examiners, and said National Board adopted a rule in substance: "State Boards are advised to not accept diplomas from colleges who graduate on one term." Diplomas were presented from the Dental Department University of Tennessee, Nashville; Dental Department of Hospital College, Louisville, Ky., and the Wisconsin Dental College, Delavin, Wis., which were granted on one term. The diplomas from the two first were on this rule only; and in justice to them and to the Indiana Board, said colleges have since complied with the requirements of the National board, and have joined the National Association of Dental Faculties. The Geo. Wilkins case—he plead that he was a graduate of the Wisconsin Dental College, and was not permitted to register as such graduate by the Board under the third Section of the law. Now the test cases have all been settled in favor of the Indiana Board of Examiners, and of the law.

There are a number of persons practicing dentistry who have not registered. Some have registered who have not had their certificates recorded, and it is desired now to reap the full benefit of the law.

If you know of any one who has not registered or recorded, notify him, or the Prosecuting Attorney without delay. If you desire blanks for Registration Certificate or Permit, write to the Secretary of the Board with stamps enclosed to insure reply, and you will be supplied.

We ask your co-operation in the execution of the law, and with our endeavors we hope to elevate the standard of dental education with an improvement in professional service to our patrons; also, the promotion of good will among practitioners, and to work to the interest of each other.

Yours Truly,

P. G. C. Hunt, M.D. D.D.S., President, Indianapolis.
R. Van Valzah, D.D.S., Terre Haute.
S. T. Kirk, D.D.S., Kokomo.
E. J. Church, D.D.S., LaPorte.
M. H. Chappell, D.D.S., Sec. and Treasurer, Knightstown.

